# Poster Session II - A190 DIAGNOSTIC UTILITY OF THE CARS SCORE FOR ACHALASIA: INTEGRATING ENDOSCOPIC FINDINGS INTO DYSPHAGIA PATHWAYS

**DOI:** 10.1093/jcag/gwaf042.190

**Published:** 2026-02-13

**Authors:** M Fida, A Bubelenyi, P Gill, Y Nasser, M Woo, C N Andrews, M Buresi, K Zhan, M Gupta

**Affiliations:** Faculty of Internal Medicine, University of Calgary, Calgary, AB, Canada; University of Calgary Cumming School of Medicine, Calgary, AB, Canada; Faculty of Internal Medicine, University of Calgary, Calgary, AB, Canada; Medicine, University of Calgary, Calgary, AB, Canada; Medicine, University of Calgary, Calgary, AB, Canada; Medicine, University of Calgary, Calgary, AB, Canada; Medicine, University of Calgary, Calgary, AB, Canada; University of Calgary Cumming School of Medicine, Calgary, AB, Canada; Medicine, University of Calgary, Calgary, AB, Canada

## Abstract

**Background:**

The diagnostic evaluation of dysphagia often requires high-resolution manometry (HRM) and EndoFLIP, which may not be widely available. The CARS score, an endoscopic assessment of achalasia features, offers a low-cost adjunct. CARS is a scoring system assessing endoscopic changes in the esophagus: Contents in esophagus, dilated Anatomy, Resistance of lower esophageal sphincter (LES) on passage of scope, and Stasis changes in the esophagus. We evaluated the role of CARS in developing a pragmatic dysphagia diagnostic pathway to streamline and minimize delay in patient care.

**Aims:**

To assess the diagnostic utility of the CARS score in predicting achalasia and to evaluate its role in developing a pragmatic dysphagia diagnostic pathway that streamlines and minimizes delays in patient care.

**Methods:**

We retrospectively analyzed 128 patients (2020–2024) who underwent EGD with abnormal HRM or EndoFLIP results. Achalasia was defined by HRM (types I–III) or EndoFLIP criteria. Associations between CARS and physiologic parameters were tested using non-parametric statistics. Predictive performance was evaluated with ROC analysis and multivariable logistic regression adjusted for age and sex.

**Results:**

Of 128 patients, 47 (36.7%) were diagnosed with achalasia. Median CARS scores were significantly higher in achalasia compared to non-achalasia patients (2 [IQR 1–2] vs. 0 [IQR 0–1]; p < 0.001). CARS correlated negatively with EndoFLIP distensibility and diameter metrics (DI at 60 cc: ρ = –0.43, p < 0.001; diameter at 60 cc: ρ = –0.51, p < 0.001). CARS was also associated with HRM markers of impaired relaxation, including pan-esophageal pressurization > 30 mmHg and rapid-drink-challenge pressurization > 20 mmHg (both p < 0.05). Receiver-operator analysis showed an AUC of 0.90, and multivariable logistic regression demonstrated that CARS independently predicted achalasia (OR 6.4, 95% CI 2.4–13.7, p < 0.001) after adjusting for age and sex.

**Conclusions:**

CARS is a simple, easily useable predictor of achalasia and can be integrated into dysphagia algorithms. At this juncture, CARS increases the pretest probability of achalasia and potentially assists expediting testing such as EndoFLIP. Incorporating CARS into the diagnostic algorithm may alter the current reliance on advanced motility testing and referrals patterns surrounding dysphagia. However, further study of these associations in a prospective manner is needed to verify these findings.

Receiver operating characteristic (ROC) curve showing diagnostic performance of the **CARS score** for predicting achalasia (AUC = 0.90, 95% CI 0.84–0.96), adjusted for age and sex.

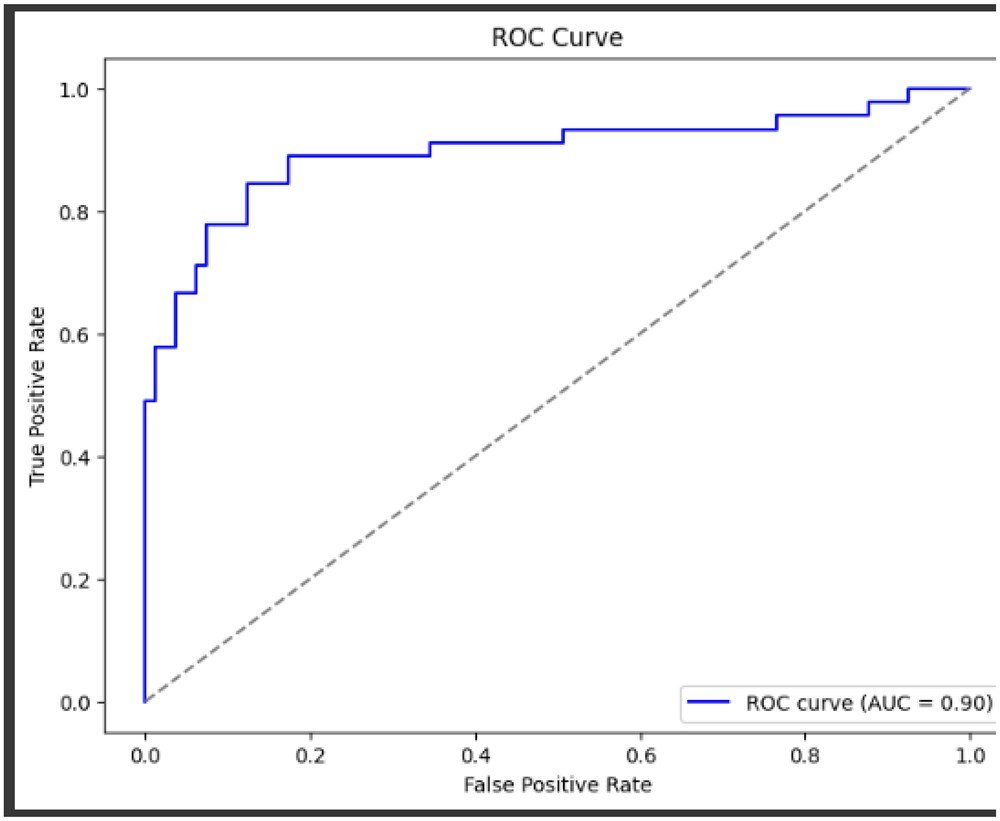

**Funding Agencies:**

None

